# Multimorbidity Patterns in Primary Care: Interactions among Chronic Diseases Using Factor Analysis

**DOI:** 10.1371/journal.pone.0032190

**Published:** 2012-02-29

**Authors:** Alexandra Prados-Torres, Beatriz Poblador-Plou, Amaia Calderón-Larrañaga, Luis Andrés Gimeno-Feliu, Francisca González-Rubio, Antonio Poncel-Falcó, Antoni Sicras-Mainar, José Tomás Alcalá-Nalvaiz

**Affiliations:** 1 IIS Aragón, Aragón Health Sciences Institute, Miguel Servet University Hospital, University of Zaragoza, Zaragoza, Spain; 2 IIS Aragón, Aragón Health Sciences Institute, San Pablo Health Centre, University of Zaragoza, Zaragoza, Spain; 3 IIS Aragón, Aragón Health Sciences Institute, South Delicias Health Centre, Zaragoza, Spain; 4 IIS Aragón, Aragón Health Sciences Institute, Primary Care Directorate of Aragón Healthcare Service, Zaragoza, Spain; 5 Planning Management, Badalona Serveis Assistencials S.A., Badalona, Spain; 6 Statistical Methods Department, University of Zaragoza, Zaragoza, Spain; University of Michigan, United States of America

## Abstract

**Objectives:**

The primary objective of this study was to identify the existence of chronic disease multimorbidity patterns in the primary care population, describing their clinical components and analysing how these patterns change and evolve over time both in women and men. The secondary objective of this study was to generate evidence regarding the pathophysiological processes underlying multimorbidity and to understand the interactions and synergies among the various diseases.

**Methods:**

This observational, retrospective, multicentre study utilised information from the electronic medical records of 19 primary care centres from 2008. To identify multimorbidity patterns, an exploratory factor analysis was carried out based on the tetra-choric correlations between the diagnostic information of 275,682 patients who were over 14 years of age. The analysis was stratified by age group and sex.

**Results:**

Multimorbidity was found in all age groups, and its prevalence ranged from 13% in the 15 to 44 year age group to 67% in those 65 years of age or older. Goodness-of-fit indicators revealed sample values between 0.50 and 0.71. We identified five patterns of multimorbidity: cardio-metabolic, psychiatric-substance abuse, mechanical-obesity-thyroidal, psychogeriatric and depressive. Some of these patterns were found to evolve with age, and there were differences between men and women.

**Conclusions:**

Non-random associations between chronic diseases result in clinically consistent multimorbidity patterns affecting a significant proportion of the population. Underlying pathophysiological phenomena were observed upon which action can be taken both from a clinical, individual-level perspective and from a public health or population-level perspective.

## Introduction

The simultaneous occurrence of various health problems in a given individual, which is defined as multimorbidity, has become the norm rather than the exception [1], [2]. Improved socio-economic and living conditions and scientific and technological advances in the field of healthcare have allowed a significant proportion of the population to survive diseases that were previously fatal, and as a result, health problems can accumulate in older population groups. A recent review conducted by Marengoni showed that the prevalence of multimorbidity in the elderly population is as high as 98% [3]. However, this phenomenon is not confined to elderly populations; its prevalence is also higher than expected for younger age groups and for both sexes [1], [4].

Understanding the types of multimorbidity patterns that appear in the elderly, especially the chronic diseases that comprise these patterns, demands an analysis of what happens in the earlier stages of life, how those patterns are shaped from a young age, in what way these diseases are aggregated, which diseases are associated with each other and which are not, as well as the pathophysiological mechanisms that may explain these associations. An improved global understanding of the disease process and the interactions present in an affected individual, rather than the study of individual diseases, is essential so that health systems can design appropriate and timely strategies for raising awareness and improving preventative and clinical approaches to multimorbidity, which currently is a health problem in and of itself [5].

Multimorbidity has extraordinary importance not only for the general population but also for healthcare systems. It has been shown to be associated with increased mortality [6], poor functional status [7], lower quality of life [8], overloaded care, especially at the primary care level [9] but also in the emergency room [10], and the greater use of specialised care [11]. Additionally, the money spent on prescription drugs has significantly increased, and there has been a greater frequency of inappropriate prescriptions as a result of this clinical and social phenomenon [9].

Despite these results, there are very few studies that have analysed multimorbidity in depth; for example, it is unknown to what degree multimorbidity is caused by chance, how multimorbidity patterns differ between age and sex groups, what the possible existence of index diseases is that would in turn give rise to new symptoms and diseases or what the roles of iatrogenic issues are in this phenomenon. Some of these aspects need to be addressed promptly.

There are three main methodological reasons for this lack of evidence:

1. First, there is uncertainty among the scientific community regarding the application of statistical methods for the exploration of simultaneously occurring health problems [6], [12]. In fact, most studies to date are based on counts of the number of health problems, or the proportion of people with pairs of conditions [13], [14]. However, a major limitation of this approach is that it cannot distinguish between statistically significant associations and those that occur simply by chance. It could be intuitively expected that diseases are either concentrated in an individual if one disease is directly responsible for the others (i.e., complicating multimorbidity) or if they all share common or correlated risk factors (i.e., causal multimorbidity) [2]. The possible existence of additional disease mechanisms beyond those of a pathophysiological nature, which make certain disease associations appear more often than expected simply by chance has also been suggested (i.e., cluster multimorbidity) [15]. Among these, socio-economic, cultural, environmental and behavioural factors have been described as related to patient complexity [16], [17].
2. Moreover, progress towards understanding the causes of multimorbidity requires a standardised conceptualisation of the various clinical entities that compose it, as the number and type of diseases together with the various ways of grouping them can significantly affect estimates of the prevalence of multimorbidity and the strength of the identified associations [18]. The interactions between chronic diseases are of particular interest from the perspective of both health and healthcare. However, serving as an additional methodological limitation, there is currently no agreed-upon systematic categorisation of chronic diseases within the scientific community.
3. Finally, the performance of large-scale population studies focused on this and other public health problems requires sources of information of proven quality that are accessible to the entire scientific community. In this regard, it is important to highlight the enormous potential of clinical-administrative databases, which are available as a result of the computerisation of medical records in both primary and specialised care. Compared with studies that have been based on survey results, these databases provide access to diagnostic information at the individual and population levels.

With respect to these three aspects, this work was based on a statistical methodology that allowed for the identification of statistically relevant patterns of multimorbidity. It applied a previously validated categorisation system for chronic diseases and used diagnostic information from primary care electronic medical records.

The primary objective of this study was to identify the existence of chronic disease multimorbidity patterns in the primary care population, describing their clinical components and analysing how these patterns change and evolve over time both in women and men. The secondary objective of this study was to generate evidence regarding the pathophysiological processes underlying multimorbidity and to understand the interactions and synergies among the various diseases.

The hypothesis was that there would be patterns of multimorbidity partially expected, predictable and explainable that evolve along patients' life. Such patterns would be composed of risk factors in the early stages of life, of organ disorders in the middle ages, and of disease-related complications in the later years of life. Healthcare systems should consider the resulting evidence when organising and planning their service offerings. Moreover, this study pretends to generate new hypotheses related to unknown causal mechanisms underlying multimorbidity such as iatrogenic effects.

## Methods

### Design, study population and variables

This multicentre, retrospective, observational study was performed in primary care centres of the Spanish National Health System. From 1986 on, Spain has a tax-based health system with universal coverage for the entire population. The primary care model is based on multidisciplinary teams consisting of family physicians and nurses. The former coordinate prevention, health promotion, treatment, and community care and they also act as gatekeepers to the healthcare system and therefore to more specialized care. Each team has a geographically delimited population assigned to it and all types of patients and health problems, except for medical emergencies, are initially attended in primary care centres.

The selection of centres to participate in this study was conducted based on the following quality inclusion criteria: (a) centres with computerised records for all appointments and with more than two years of experience using this system by all physicians and nurses, (b) those with a percentage of uncoded episodes less than 20%, (c) those with a percentage of notes (e.g., prescription, observation, etc.) listed in uncoded episodes less than 15%, (d) those with a percentage of prescriptions linked to uncoded episodes less than 10%, (e) those with an average number of diagnoses higher than 3.5, and (f) those with a percentage of patients with no diagnostic information less than 10%.

After selecting the centres, all patients over the age of 14 who were seen at least once during 2008 by their family doctor were included in the study. The study population was composed of 275,682 individuals belonging to 19 urban health centres (7 in Aragón and 12 in Catalonia). For each of the patients included in the study, demographic and diagnostic variables were extracted from electronic medical records, including the age of the patient as of December 31, 2008, the sex of the patient, and the chronic diagnoses coded according to the International Classification of Primary Care (ICPC) [19]. The diagnostic data included a comprehensive overview of patients' chronic diseases assigned until end of 2008, for which there had been an annotation in the medical record (e.g., prescription, observation, etc.) at some point during the study year (i.e., 2008).

To facilitate the management of the diagnostic information, the episodes were grouped according to the Expanded Diagnostic Clusters (EDC) of the ACG® system. To use this system, a conversion from the ICPC to the International Classification of Diseases (ICD-9-CM) [20] was made beforehand, using a mapping method developed by the research team. Thus, the ACG® system grouped each ICD-9-CM code as a single EDC from a total of 264 EDCs in the system, based on the clinical, diagnostic and therapeutic similarities of the diseases [21].

The selection of which chronic diseases to study was based on the recent study by Salisbury et al. [22]. Here, a chronic disease was defined as one whose duration was 6 months or more, including past conditions that required continuing care, major diseases with a recurrence risk and/or past diseases with continued implications for patient management (Table S1).

This study was favourably evaluated by the Clinical Research Ethics Committee of Aragon (CEICA). Written consent by patients was not needed since the study does not involve interventions on individuals, the use of human biological samples, or the analysis of personally identifiable data. Instead, the present work is based on the statistical analysis of anonymous data contained in previously existing databases which were obtained with prior permission from the corresponding entity.

### Statistical analysis

#### Descriptive analysis

A descriptive analysis of the population was performed by calculating the frequencies of demographic variables. To facilitate the analysis of age, three age ranges were created: 15–44, 45–64 and 65 and over. The remaining analyses were performed separately for each age and sex group. To provide an initial description of the prevalence of multimorbidity, the frequency of EDCs per patient for each group of age and sex was calculated.

#### Factor analysis

To determine the associations between diseases that exhibited multimorbidity patterns, an exploratory factor analysis was applied. The aim of this method was to identify sets of variables with a common underlying causal factor. This technique was chosen because, in addition to identifying associations among groups of variables, it allowed for variables to be included in the various patterns.

To increase the epidemiological interest of the study, only those EDCs with a prevalence equal to or greater than 1% in each group were included (Table S1). These EDCs were coded in binary format (i.e., 0 = no disease and 1 = presence of the disease). The factor analysis was based on a correlation matrix to determine which diagnostic variables comprised each factor. Due to the dichotomous nature of the variables, tetra-choric correlation matrices were used [23]. In doing so, it was assumed that dichotomous diagnoses had underlying, continuous characteristics. That is, we assumed that the chronic diseases included in our analysis had a progressive course (i.e., the accumulation of risk factors before the onset and/or the progression after the onset of the disease) and were diagnosed during this course if they reached a certain threshold. The factors resulting from this analysis were interpreted as multimorbidity patterns (i.e., EDCs frequently related to each other), and each factor score, which had a value between −1 and 1, represented the association of each of the diagnoses with its disease pattern.

The extraction of the disease factors was performed using the principal factor method, and it was assumed that the extracted factors did not explain the total variance of the analysed EDCs. To determine the number of factors to extract, a scree plot was utilised in which the eigenvalues of the correlation matrix were represented in descending order. The number of factors extracted corresponds to the sequence number of the eigenvalue that produces the inflection point of the curve [24]. In some situations, the Heywood phenomenon was observed due to the uniquely negative scores, which produced factor scores greater than 1. This can occur when a small or excessive number of factors are removed. In this case, as well as when a clear solution was not obtained by the scree plot, a clinical evaluation of different solutions was conducted. To facilitate the interpretation of the factors, an oblique rotation (Oblimin) was applied.

The adequacy of the sample used to perform the factor analysis was analysed by measuring the Kaiser-Meyer-Olkin (KMO) for each age and sex group. This parameter takes values between 0 and 1, which are closer to 1 with a greater goodness of fit. In addition, as a measure of model goodness-of-fit, the proportion of cumulative variance was obtained, which describes the variability of the diagnostic data explained by the patterns.

For determining which EDCs formed each multimorbidity pattern, those with scores equal to or greater than 0.25 for each factor were selected.

Given that multimorbidity is defined as the simultaneous presence of two or more chronic diseases [1], it was agreed that an individual had a specific pattern of multimorbidity if he/she presented with at least two of the diseases that comprised the pattern. Ultimately, this permitted the calculation of the prevalence of each of the multimorbidity patterns.

STATA 11.0 software was used to conduct the statistical analysis, and Excel 2007 was used to prepare the graphs.

### Clinical consistency of the patterns

Clinical relevance was studied in three phases. First, two qualified primary care physicians with research and clinical experience independently reviewed the clinical plausibility of disease interactions. Next, a joint analysis of both independent reports was performed by a third physician specialized in public health and experienced in health services research, identifying agreements and disagreements between both family physicians. Last, results were resubmitted to the primary care professionals and discussed in a final consensus meeting. The main arguments and conclusions were also contrasted with a literature review by other members of the research team.

## Results

### Descriptive analysis

**Figure 1:**
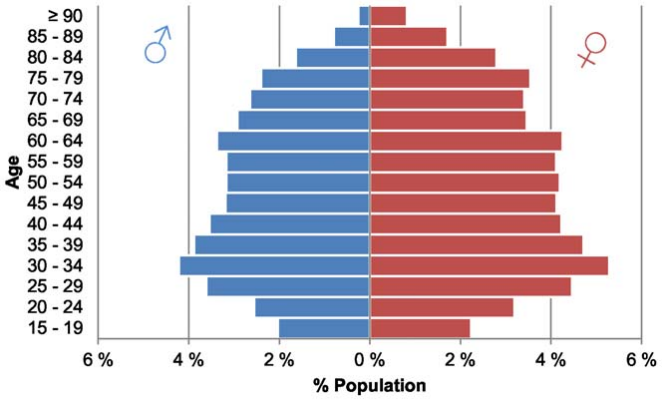
Population pyramid, year 2008.

The study population consisted of 275,682 patients over the age of 14, and 56.6% of this population was female. Figure 1 shows the population pyramid, and Table 1 shows the population distribution of the six age and sex groups.

**Table 1:** Population distribution according to age and sex.

	15–44 years		45–64 years		≥65 years	
	n	%	n	%	n	%
Men	54,705	19.8	35,587	12.9	29,361	10.7
Women	66,540	24.1	46,035	16.7	43,454	15.8

Multimorbidity existed in all of the studied groups (Table 2). An increasing number of simultaneous diseases in an individual were observed as age increased, and there were no apparent gender differences. In younger people aged 15 to 44, 13% had more than two simultaneous chronic diseases (11% men and 16% women), and for patients between 45 and 64 years of age, this figure rose to 43% (39% men and 47% women). Furthermore, this percentage reached 67% in the group of individuals 65 years of age or older (65% men and 69% women).

**Table 2:** Distribution of the number of chronic EDCs within each age and sex group.

	15–44 years				45–64 years				≥65 years			
	Men		Women		Men		Women		Men		Women	
N^o_^ chronic EDCs	N	%	n	%	n	%	n	%	n	%	n	%
0	32,422	59.27	34,824	52.34	9,940	27.93	10,413	22.62	3,486	11.87	4,642	10.68
1	16,293	29.78	20,911	31.43	11,683	32.83	14,021	30.46	6,855	23.35	8,687	19.99
2	4,442	8.12	7,403	11.13	7,291	20.49	10,065	21.86	6,948	23.66	9,703	22.33
3	1,132	2.07	2,370	3.56	3,871	10.88	6,140	13.34	5,345	18.2	8,306	19.11
4	299	0.55	737	1.11	1,703	4.79	3,075	6.68	3,425	11.67	5,775	13.29
5	85	0.16	205	0.31	687	1.93	1,406	3.05	1,785	6.08	3,374	7.76
6	23	0.04	76	0.11	250	0.7	575	1.25	829	2.82	1,649	3.79
7	5	0.01	10	0.02	102	0.29	214	0.46	414	1.41	759	1.75
8	3	0.01	4	0.01	46	0.13	80	0.17	169	0.58	311	0.72
9	1	0	–	–	10	0.03	36	0.08	74	0.25	153	0.35
10	–	–	–	–	3	0.01	7	0.02	17	0.06	64	0.15
11	–	–	–	–	–	–	2	0	11	0.04	21	0.05
12	–	–	–	–	1	0	1	0	1	0	6	0.01
13	–	–	–	–	–	–	–	–	–	–	4	0.01
14	–	–	–	–	–	–	–	–	2	0.01	–	–
Total	54,705	100	66,540	100	35,587	100	46,035	100	29,361	100	43,454	100

### Multimorbidity Patterns

#### Men 15 to 44 years old

**Figure 2:**
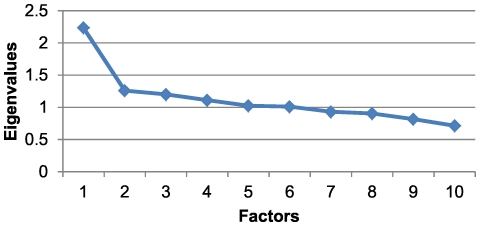
Scree plot for men in the 15 to 44 age range.

This age and sex group had a KMO sampling adequacy index of 0.66 and a cumulative variance percentage of 26.87% (Table 3). The scree plot for this group indicated that the number of factors extracted was equal to two, as the point of inflection on the curve occurred at the eigenvalue occupying that position (Figure 2). The first factor was determined by the association between hypertension, diabetes, obesity and lipid metabolism disorders. The second factor was composed of substance abuse, anxiety and neurosis, schizophrenia and affective psychosis, lipid metabolism disorders and obesity (Table 4). In this group, 2.07% of the individuals belonged to at least one of the two patterns that were obtained, and there was a prevalence of 0.93% for the first pattern and 1.53% for the second.

**Table 3:** KMO values and the % of accumulated variance of the factor analysis carried out within each age and sex group.

	15–44 years		45–64 years		≥65 years	
	Men	Women	Men	Women	Men	Women
KMO	0.66	0.71	0.50	0.68	0.57	0.68
% Accumulated Variance	26.87	24.72	14.80	23.70	22.81	24.68

**Table 4:** Factor score for each EDC in men 15 to 44 years of age.

Chronic EDCs	Factor1	Factor2
**Disorders of lipoid metabolism**	**0.42**	**0.29**
**Hypertension**	**0.65**	0.13
**Diabetes**	**0.46**	0.05
**Obesity**	**0.45**	**0.26**
**Anxiety, neuroses**	0.03	**0.39**
**Substance use**	−0.08	**0.53**
**Schizophrenia and affective psychosis**	0.01	**0.36**
Asthma, w/o status asthmaticus	−0.10	0.04
Thyroid disease	0.14	0.15
Arthropathy	0.12	−0.03
Cervical pain syndromes	0.14	0.02
Low back pain	0.04	−0.02
Dermatitis and eczema	0.09	0.03

Note: factor scores ≥0.25 have been highlighted in bold.

#### Men 45 to 64 years old

**Figure 3:**
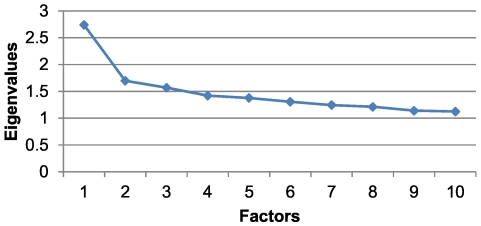
Scree plot for men in the 45 to 64 age range.

For this age and sex group, a KMO value of 0.50 was obtained with a cumulative variance percentage of 14.80% (Table 3). The scree plot again pointed to the relevance of extracting two factors (Figure 3). The first factor was similar to the first factor that was found for men aged 15–44 but also included other diseases, such as ischemic heart disease, cardiac arrhythmia, atherosclerosis, and acute myocardial infarction. The second factor comprised obesity, arthropathy, back pain, varicose veins in the lower extremities and gastro-oesophageal reflux (Table 5). In this group, 13.61% of the individuals belonged to at least one of the two patterns obtained, and there was a prevalence of 9.20% for the first pattern and 4.86% for the second.

**Table 5:** Factor score for each EDC in men 45 to 64 years of age.

	Factor1	Factor2
**Ischemic heart disease (excluding infarction)**	**0.35**	−0.08
**Cardiac arrhythmia**	**0.44**	−0.08
**Generalised atherosclerosis**	**0.45**	−0.01
**Acute myocardial infarction**	**0.30**	−0.03
**Hypertension**	**0.42**	0.14
**Diabetes**	**0.46**	−0.07
**Chronic liver disease**	**0.33**	−0.06
**Obesity**	**0.27**	**0.26**
**Substance use**	**0.34**	−0.14
**Emphysema, chronic bronchitis, COPD**	**0.36**	0.09
**Deafness, hearing loss**	−0.08	**0.26**
**Gastro-oesophageal reflux**	−0.05	**0.34**
**Varicose veins of lower extremities**	0.01	**0.29**
**Prostatic hypertrophy**	0.08	**0.30**
**Arthropathy**	0.02	**0.25**
**Low back pain**	−0.02	**0.28**
Disorders of lipoid metabolism	0.24	0.17
Haematologic disorders, other	0.21	0.02
Asthma, w/o status asthmaticus	−0.08	0.23
Cervical pain syndromes	−0.01	0.22
Dermatitis and eczema	0.05	0.23
Cardiovascular disorders, other	0.19	0.02
Thyroid disease	0.19	0.06
Glaucoma	0.10	0.10
Renal calculi	0.06	0.15
Peripheral neuropathy, neuritis	0.09	0.12
Anxiety, neuroses	0.17	0.10
Schizophrenia and affective psychosis	0.02	−0.08
Gout	0.16	0.17
Psoriasis	0.04	0.06

Note: factor scores ≥0.25 have been highlighted in bold.

#### Men 65 years of age or older

**Figure 4:**
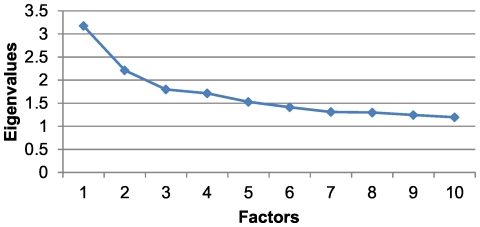
Scree plot for men older than 65 years of age.

For this age and sex group, a KMO value of 0.57 was obtained with a cumulative variance percentage of 22.81% (Table 3). Although the scree plot indicated that it would be appropriate to extract 4 factors (Figure 4), the Heywood phenomenon was observed, and it was therefore decided that 3 factors would be extracted. The first factor was similar to the first factors found for men 15–44 and 45–64 years of age but also included conditions such as congestive heart failure, anaemia and gout. In addition, there was a new factor associated with osteoporosis, Parkinson's disease, dementia/delirium and skin ulcers. Moreover, the second factor for men aged 45–64, which was now termed Factor 3, was present for this group and incorporated new diseases, such as cervical pain, dermatitis and eczema (Table 6). In this group, 30.99% of the individuals belonged to at least one of the three patterns obtained, and the prevalence of Factor 1 was 20.25%, that of Factor 2 was 1.67% and that of Factor 3 was 13.56%.

**Table 6:** Factor score in each EDC in men 65 years of age and older.

	Factor1	Factor2	Factor3
**Congestive heart failure**	**0.52**	**0.25**	0.03
**Cardiac arrhythmia**	**0.34**	0.19	0.09
**Generalised atherosclerosis**	**0.28**	0.17	0.08
**Hypertension**	**0.34**	−0.10	0.09
**Diabetes**	**0.36**	0.03	−0.09
**Iron deficiency, other deficiency anaemias**	**0.29**	**0.33**	0.15
**Haematologic disorders, other**	**0.35**	−0.07	0.01
**Obesity**	**0.29**	−0.22	0.04
**Gout**	**0.27**	−0.08	0.03
**Cardiovascular disorders, other**	**0.25**	0.05	0.00
**Osteoporosis**	−0.21	**0.35**	**0.40**
**Parkinson's disease**	0.02	**0.35**	0.00
**Dementia and delirium**	−0.08	**0.38**	0.10
**Behaviour problems**	0.04	**0.28**	0.04
**Chronic ulcer of the skin**	0.23	**0.59**	−0.15
**Cerebrovascular disease**	0.20	**0.25**	0.00
**Gastro-oesophageal reflux**	−0.06	0.09	**0.30**
**Prostatic hypertrophy**	−0.07	−0.04	**0.31**
**Arthropathy**	0.03	−0.02	**0.29**
**Cervical pain syndromes**	−0.03	−0.06	**0.29**
**Low back pain**	−0.01	−0.02	**0.36**
**Anxiety, neuroses**	0.03	0.20	**0.28**
**Dermatitis and eczema**	0.00	0.04	**0.32**
Acute myocardial infarction	0.23	−0.02	−0.06
Ischemic heart disease (excluding infarction)	0.20	0.04	0.18
Thyroid disease	0.06	0.16	0.20
Emphysema, chronic bronchitis, COPD	0.15	0.12	0.24
Malignant neoplasms, colorectal	0.07	0.07	0.00
Disorders of lipoid metabolism	0.09	−0.17	0.17
Malignant neoplasms, prostate	−0.03	0.19	−0.02
Peripheral neuropathy, neuritis	0.19	0.01	0.15
Cataract, aphakia	0.09	−0.05	0.19
Glaucoma	0.04	0.00	0.11
Psoriasis	0.04	−0.08	0.11
Deafness, hearing loss	−0.04	−0.11	0.14
Varicose veins of lower extremities	0.05	0.05	0.19
Low impact malignant neoplasms	0.00	0.05	0.14
Asthma, w/o status asthmaticus	−0.12	−0.08	0.18

Note: factor scores ≥0.25 have been highlighted in bold.

#### Women 15 to 44 years old

**Figure 5:**
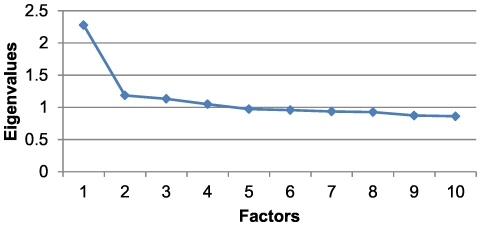
Scree plot for women in the 15 to 44 age range.

The sampling adequacy of this group had a KMO value of 0.71 with a cumulative variance percentage of 24.72% (Table 3). The inflection of the scree plot corresponded to two factors (Figure 5). The first factor, which had a prevalence of 0.38%, was composed of obesity, hypertension and lipid metabolism disorders. The second factor, which had a prevalence of 2.69%, was composed of varicose veins in the legs, iron deficiency anaemia, arthropathy, neck pain, behavioural problems (insomnia), dermatitis and eczema and diseases of the hair and follicles (Table 7). Overall, 3.03% of women between 15 and 44 years of age belonged to one of these two patterns.

**Table 7:** Factor score of each EDC in women between 15 and 44 of years of age.

	Factor1	Factor2
**Disorders of lipoid metabolism**	**0.33**	0.16
**Hypertension**	**0.60**	0.04
**Obesity**	**0.56**	0.09
**Varicose veins of lower extremities**	−0.02	**0.36**
**Iron deficiency, other deficiency anaemia**	0.03	**0.30**
**Arthropathy**	0.09	**0.26**
**Cervical pain syndromes**	−0.04	**0.32**
**Behaviour problems**	0.04	**0.27**
**Dermatitis and eczema**	0.03	**0.29**
**Disease of hair and hair follicles**	−0.09	**0.26**
**Thyroid disease**	0.14	**0.25**
Low back pain	0.05	0.22
Anxiety, neuroses	0.10	0.22
Asthma, w/o status asthmaticus	0.18	−0.02

Note: factor scores ≥0.25 have been highlighted in bold.

#### Women 45 to 64 years old

**Figure 6:**
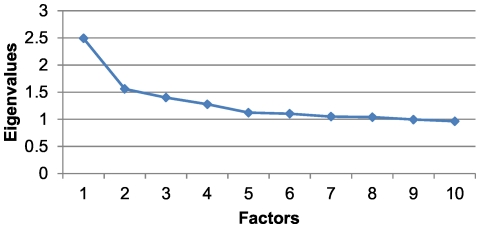
Scree plot for women in the 45 to 64 age range.

The sampling adequacy of this group had a KMO value of 0.68 with a cumulative variance percentage of 23.70% (Table 3). The scree plot clearly demonstrated that there was no ideal place at which to set the cut-off point (Figure 6). As a result, a clinical interpretation of the various options was conducted, and it was decided that three factors should be extracted. The first factor, which had a prevalence of 4.05%, was similar to Factor 1 obtained for women aged 15–44 but diabetes replaced the lipid metabolism disorders. The second factor, which had a prevalence of 11.69%, was composed of thyroid disease, gastro-oesophageal reflux, varicose veins in lower extremities, neck pain, back pain, anxiety and neurosis, osteoporosis and dermatitis and eczema. This factor was partially coincident with Factor 2 from the group of women aged 15–44. The third factor, which had a prevalence of 0.12%, was composed of behavioural problems (insomnia) and depression (Table 8). Overall, 15.01% of women between 45 and 64 years of age belonged to one of these three patterns.

**Table 8:** Factor score of each EDC in women between 45 and 64 years of age.

	Factor1	Factor2	Factor3
**Hypertension**	**0.77**	−0.11	0.02
**Diabetes**	**0.58**	−0.10	0.02
**Obesity**	**0.37**	0.16	0.03
**Thyroid disease**	0.07	**0.29**	−0.16
**Gastro-oesophageal reflux**	0.05	**0.40**	0.04
**Varicose veins of lower extremities**	0.01	**0.26**	0.07
**Cervical pain syndromes**	−0.07	**0.29**	0.10
**Low back pain**	0.02	**0.28**	0.16
**Anxiety, neuroses**	−0.01	**0.34**	−0.16
**Osteoporosis**	−0.03	**0.25**	−0.14
**Dermatitis and eczema**	0.02	**0.25**	0.05
**Behaviour problems**	−0.02	0.20	**0.29**
**Depression**	−0.01	0.06	**0.68**
Asthma	0.03	0.18	−0.01
Disorders of lipoid metabolism	0.21	0.19	−0.08
Cardiovascular disorders, other	0.07	0.17	0.02
Other endocrine disorders	0.05	0.03	0.21
Glaucoma	0.16	0.12	0.11
Iron deficiency, other deficiency anaemia	−0.02	0.17	−0.02
Haematologic disorders, other	0.08	0.13	−0.06
Low impact malignant neoplasms	0.00	0.12	−0.33
Arthropathy	0.09	0.21	0.19
Peripheral neuropathy, neuritis	0.03	0.23	0.06

Note: factor scores ≥0.25 have been highlighted in bold.

#### Women 65 years of age or older

**Figure 7:**
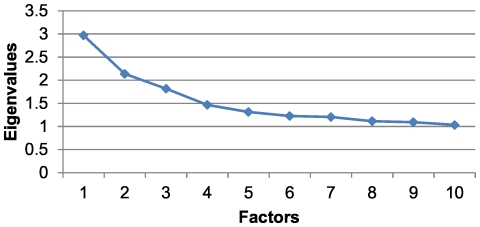
Scree plot for women older than 65 years of age.

The sampling adequacy of this group had a KMO value of 0.68 with a cumulative variance percentage of 24.68% (Table 3). The scree plot indicated that it was appropriate to extract four factors (Figure 7). The first factor, which had a prevalence of 33.30%, was very similar to Factor 1 for women aged 45–64 but also included diseases such as osteoporosis, cervical pain and varicose veins in the lower extremities. The second factor, which had a prevalence of 3.84%, was composed of congestive heart failure, cardiac arrhythmia, iron deficiency anaemia, cerebrovascular disease, dementia/delirium and skin ulcers. The third factor (prevalence of 17.30%) consisted of the association of ischemic heart disease, hypertension, diabetes, obesity, haematological problems, cardiac arrhythmia and congestive heart failure, and this coincided with Factor 1 for the groups of women aged 15–44 and 45–64. In addition, the factor that was composed of behavioural problems (insomnia) and depression (Table 9) was also seen for this age group (prevalence of 0.16%), as it had been for women aged 45–64. Overall, 45.34% of women 65 years of age or older belonged to at least one of these four patterns.

**Table 9:** Factor score of each EDC in women 65 years of age and older.

	Factor1	Factor2	Factor3	Factor4
**Disorders of lipoid metabolism**	**0.25**	−0.16	0.04	−0.06
**Osteoporosis**	**0.40**	−0.07	−0.17	−0.07
**Thyroid disease**	**0.25**	0.01	0.02	−0.13
**Gastro-oesophageal reflux**	**0.44**	0.13	−0.14	0.09
**Diverticular disease of colon**	**0.31**	0.14	−0.08	0.01
**Varicose veins of lower extremities**	**0.30**	0.03	0.05	−0.02
**Arthropathy**	**0.30**	−0.04	0.09	0.00
**Cervical pain syndromes**	**0.28**	−0.09	−0.02	0.01
**Low back pain**	**0.34**	−0.06	0.05	0.07
**Anxiety, neuroses**	**0.37**	0.18	−0.07	−0.13
**Dermatitis and eczema**	**0.27**	0.02	0.03	0.04
**Congestive heart failure**	−0.04	**0.39**	**0.37**	0.03
**Cardiac arrhythmia**	−0.02	**0.34**	**0.36**	−0.14
**Iron deficiency, other deficiency anaemia**	0.10	**0.35**	0.14	0.00
**Cerebrovascular disease**	−0.03	**0.36**	0.08	0.08
**Dementia and delirium**	−0.01	**0.42**	−0.13	0.00
**Chronic ulcer of the skin**	−0.16	**0.50**	0.10	0.02
**Ischemic heart disease (excluding infarction)**	0.09	0.18	**0.29**	−0.06
**Hypertension**	0.11	−0.08	**0.44**	0.03
**Diabetes**	−0.13	0.04	**0.46**	−0.02
**Haematologic disorders, other**	0.01	0.12	**0.31**	0.05
**Obesity**	0.13	−0.25	**0.30**	0.13
**Behaviour problems**	0.17	0.19	−0.06	**0.39**
**Depression**	0.06	−0.06	0.05	**0.79**
Asthma, w/o status asthmaticus	0.23	−0.02	0.12	−0.01
Cataract, aphakia	0.22	−0.01	0.06	0.09
Glaucoma	0.20	−0.01	0.07	0.02
Generalised atherosclerosis	0.07	0.23	0.09	−0.14
Cardiovascular disorders, other	0.07	0.10	0.21	0.09
Deafness, hearing loss	0.17	0.03	0.02	0.13
Peripheral neuropathy, neuritis	0.15	−0.05	0.10	−0.17
Emphysema, chronic bronchitis, COPD	0.15	0.18	0.05	0.00
Low impact malignant neoplasms	0.11	0.10	−0.05	−0.33
Other endocrine disorders	0.10	0.00	0.14	0.18

Note: factor scores ≥0.25 have been highlighted in bold.

Most of the studied diseases were present in one single pattern, and only 6 of the 37 diseases that comprised the 5 patterns were associated with more than one pattern; these included lipid metabolism disorders (in men), obesity (in men), congestive heart failure (in women and men), osteoporosis (in men), cardiac arrhythmia (in women) and iron deficiency (in men).

## Discussion

This study revealed five specific clinically consistent patterns of multimorbidity in the adult population: cardio-metabolic, psychiatric-substance abuse, mechanical-obesity-thyroidal, psychogeriatric and depressive. Two of them were found to evolve throughout life and differed in their presentation for men and women (i.e., cardio-metabolic and mechanical-obesity-thyroidal), three of them affected both sexes (i.e., cardio-metabolic, mechanical-obesity-thyroidal and psychogeriatric), and two of them were present exclusively in men or women (i.e., psychiatric-substance abuse and depressive, respectively) (Table 10).

**Table 10:** Identified multimorbidity patterns and their prevalence within each age and sex group.

	Men		Women	
	Multimorbidity pattern	Prevalence (%)	Multimorbidity pattern	Prevalence (%)
15–45 years	Cardio-metabolic	0.93	cardio-metabolic	0.38
	psychiatric-substance abuse	1.53	mechanical-obesity-thyroidal	2.69
45–64 years	Cardio-metabolic	9.20	cardio-metabolic	4.05
	mechanical-obesity-thyroidal	4.86	mechanical-obesity-thyroidal	11.69
			depressive	0.12
≥65 years	Cardio-metabolic	20.25	cardio-metabolic	33.30
	mechanical-obesity-thyroidal	1.67	mechanical-obesity-thyroidal	3.48
	psychogeriatric	13.56	psychogeriatric	17.30
			depressive	0.16

Those two patterns suggesting an evolution throughout life were composed mainly of risk factors in the youngest age group, of organ disorders in the middle-aged group, and of various disease-related complications in the eldest group.

Although there are many examples in the literature of the association between the specific diseases that comprise these patterns, there have been very few population-wide published studies on multimorbidity including the non-elderly population.

### Multimorbidity Patterns

#### Cardio-metabolic

This multimorbidity pattern was present in both sexes and for all of the age ranges analysed. Although it was especially prevalent among individuals 65 years of age or older (i.e., one in three women and one out of every five men in this age group), the high prevalence of this pattern in the middle-aged groups should be noted (i.e., 22% in women and about 10% in men).

Its development in men and women was consistent with the disease pathophysiology. In young patients, it was manifested as diabetes, hypertension, obesity and dyslipidaemia [25], and its progression over time was associated with the expected complications for this type of diseases, which were primarily cardiac in nature.

In men, this pattern presented at a young age and with a possible common pathophysiological basis of insulin resistance, obesity and their associated inflammatory processes, and negative lifestyles (e.g., physical inactivity, poor diet, etc.). During middle age, ischemic heart disease, atherosclerosis, myocardial infarction, arrhythmias, dyslipidaemia (with a factor score very close to the established cut-off point), substance abuse, COPD and chronic liver disease were also observed within this pattern. Although this study did not have access to specific information regarding tobacco smoking, it is likely that its prevalence was high in those cases where the abuse of other substances was also high (this information was available). The alcohol-tobacco association could have been the probable cause of the diseases observed, such as chronic liver disease and other related associations. In addition, there are possible underlying iatrogenic causes due to the use of drugs such as statins and fibrates, which can cause the elevation of transaminases at medium and high dosages [26]. Dyslipidaemia was present in middle-aged individuals but was absent in men 65 years of age or older, which is inconsistent with the clinical picture observed for this age group. It is likely that this absence was due to an underreporting bias, given the greater relative severity of other diseases in this group. The presence of heart failure in the group of 65 years and over could be seen as the final link in the natural history of this cardio-metabolic disease. In our study, gout was added to this pattern, and its association with metabolic syndrome has recently been demonstrated [27]–[29].

The presence and the evolution of this cardio-metabolic pattern revealed differences between women and men. In women, although hypertension, obesity and dyslipidaemia were present at a young age, diabetes did not appear until the age range 45–64. Moreover, in this second age group, the expected cardiac complications were not observed, contrary to what happened in men of the same age. Oestrogenic protection and reduced smoking have been previously reported as the primary reasons for reduced cardiac complications in women [30]. We propose a hypothesis for this study, which has also been examined by other authors [31]–[33], holding that metabolic syndrome should not be considered as a single, whole disease but rather that the obesity component should be considered the index factor, which when present at an early age of life, activates the immune system in both men and women and leads to the development of the so-called metabolic syndrome. Type 2 diabetes, therefore, would not be part of the “whole” (metabolic syndrome) but would rather be the consequence of the initial condition (obesity). This, together with the protective effect of oestrogen, would be the reason why Type 2 diabetes appeared in women after the age of 45 in this study. However, in the oldest age group studied (65 years of age and over), this pattern was presented similarly in men and women, amplifying this syndrome along with the various manifestations of heart disease, including ischemic heart disease, arrhythmias and congestive heart failure. Additionally, dyslipidaemia was absent in this group, as observed in men of this age, and this was likely due to the disease recording habits of professionals. In women, heart failure may have also served as the final link in the natural history of this cardio-metabolic pattern.

#### Psychiatric – Substance abuse

This multimorbidity pattern appeared only in young men and was found to affect 2% of the individuals studied (n = 837). This pattern consisted of psychopathological processes, such as psychosis and neurosis, which are both likely related to the toxic substance abuse that is also present within this pattern and that commonly affects men at this stage of life [34], [35]. Psychoactive drugs may act as an agglutinative factor leading to psychosis, which in its early stages favours substance abuse [36], [37]. This clinical picture also involves obesity, which is a consequence of this poor lifestyle as well as the use of new-generation antipsychotic medications [38]. This causal hypothesis is supported by the fact that this pattern did not appear among women, where toxic substance abuse occurs less frequently [35].

#### Mechanical

In men, this was a complex pattern that could also be denominated mechanical-obesity-thyroidal. It affected 5% of middle-aged individuals and was found to decrease to 2% among individuals aged 65 and over. For men aged 45–64, it is likely that obesity acted as an index disease that favoured the emergence of mechanical disorders due to excess body weight, such as arthropathy, cervical and low back pain, varicose veins of lower extremities, and gastro-oesophageal reflux [39]–[41]. An increased risk for benign prostatic hypertrophy has also been found in obese men [42]. However, this hypothesis should be further supported since, unexpectedly, obesity does not appear in men over 65. Instead, the factor score for thyroid problems became very close to the established cut-off point in this age group. The latter could explain some of the associations and interactions between the various diseases found within this pattern. It should finally be mentioned that for patients 65 years of age or older, this pattern shared clear similarities with fibromyalgia [43].

In women, the mechanical pattern was manifest somewhat differently than in men. It appeared early in life and it was never associated with obesity but rather with thyroid problems, likely in the form of hypothyroidism [44], [45]. While the prevalence of this pattern was 3% among women of the 15 to 44 age group, it was very high among women aged 45 to 64 affecting more than one in five women (22%). At this age, the pattern was amplified by neurogenic disorders, such as anxiety and dermatitis. Finally, for women in the 65 and over age group, the pattern was presented in its most amplified state (i.e. arthropathy, cervical and low back pain, varicose veins of lower extremities, gastro-oesophageal reflux, osteoporosis and neurogenic disorders) and was associated with various other disorders, such as dyslipidaemia or diverticulosis, for which the association is difficult to explain according to available knowledge.

#### Psychogeriatric

This multimorbidity pattern appeared in individuals 65 years of age and older and was the second-most prevalent pattern after cardio-metabolic. It was more common in women (17%) than men (14%) and was manifest in the form of dementia, behavioural problems, Parkinson's disease, osteoporosis, chronic skin ulcers and iron deficiency. Heart failure and stroke showed factor scores very close to the established cut-off-point and are among the leading causes of dementia [46]. Dementia can also be caused by Parkinson's disease, and the antipsychotic treatment of the behavioural disturbances in patients with dementia can cause Parkinsonism [47].

The presence of dementia and Parkinson's disease together with age-related osteoporosis can lead to falls, fractures, the immobility of the patients and the appearance of skin ulcers caused by bed rest among these patients [48].

In women, this pattern was slightly different than in men: dementia, cerebrovascular disease and chronic skin ulcers were present. In addition, heart failure, iron deficiencies and cardiac arrhythmia (mostly in the form of atrial fibrillation) were present in this pattern, and these were clearly associated with and were the cause of cerebrovascular disease [49]. Parkinson's disease, osteoporosis and behavioural problems disappeared in women with respect to men.

#### Depressive

This multimorbidity pattern consisted of only two conditions: depression and behavioural disorders (mainly insomnia). These were strongly associated with one another and were present only in women aged 45–64 and 65 years and over. This was the least prevalent of the five patterns described, as the frequency was less than 0.2% in both age groups. The association between these two clinical conditions has been widely described [50], and it has also been shown that untreated anxiety disorders can develop into depression [51]. It is noteworthy that anxiety was present in our study population as part of the thyroid pattern in both men and women.

### Strengths

The two main aspects that influence result-stability are the nature and number of individuals and diseases that are included in the analysis [52]. The diagnoses in this study were taken from the electronic medical records of 275,682 primary care adult patients in the context of a national health system with universal coverage, which has been shown to lead to more reliable and representative conclusions compared to those derived from survey-based studies [53].

Regarding the statistical methodology used, the exploratory factor analysis applied in this study is the preferred method when the objective is to explore statistically significant stable disease clusters [54]. In the absence of inferential statistics on which to base the analytical choices related to this type of method, the decisions were based on a set of rules and recommendations for the social sciences that have previously been described by Costello et al. [55]. Thus, this study adhered to the following criteria: (a) the use of the principal factor method as the technique for extracting factors by assuming a nonparametric distribution of binary data (presence/absence of disease); (b) the use of scree plots together with a clinical assessment of the results to select the number of factors; (c) the oblique rotation of factors; (d) factor scores greater than 0.30, which is set as the minimum acceptable value for the correlation to be significant from a clinical and statistical perspective; (e) the detection of few or no diseases with strong associations with several factors; (f) the identification of at least three diseases per factor; (g) obtaining samples from more than 100 individuals and (h) the presentation of the factor scores.

It is noteworthy that this study met most of these standards. The decision to reduce the minimum factor score to 0.25 (or even 0.20) was due to the expectation that there would be a significant number of associations among diseases due to chance (i.e., concurrent multimorbidity). Therefore, a more permissive threshold was established. On the other hand, despite the fact that the depressive pattern comprised only two diseases (depression and behavioural disorders, such as insomnia), this pattern has been previously identified in the literature [56]. Moreover, the goodness-of-fit values for the models, expressed as the percentage of the accumulated variance (i.e., between 14.80% and 26.87%) and the sampling adequacy (i.e., KMO measures between 0.50 and 0.71) were above the acceptable lower limits.

### Limitations

Although several hypothesis have been put forward on the pathophysiological processes underlying the five multimorbidity patterns brought to light in this study, the former must be interpreted with the necessary caution since the study design (i.e. transversal) does not allow to establish the sequence in which diseases cluster within a pattern. Longitudinal studies would be required to corroborate the suggested causal associations as well as to help elucidate those disease associations that could not be explained in the present study.

As stated earlier, this study was based on information that was recorded in the primary care electronic medical record system during medical visits. Although there are many benefits of this methodology, it can also limit the data. The workload of healthcare professionals and the structure of the applied diagnostic coding system (i.e. ICPC) often lead certain information regarding disease history not to be recorded in the individual patient medical histories. Therefore, the frequencies of many diseases are often underestimated. We suspect that this may have occurred for smoking, which is systematically underreported in a population with a very high prevalence of smokers [57]. This led us to exclude this information from the analysis and therefore prevented us from providing a plausible explanation to some of the associations found with the cardio-metabolic pattern. However, the fact that we focused this study on chronic diseases, which have diagnostic codes that remain in the health record over time, may have helped to minimise this problem. Furthermore, this constraint is expected to be minimal, given the quality criteria that were used to select the centres included in the study.

A potential overrepresentation of certain diagnoses may exist when these are associated with other diseases for which treatment protocols recommend periodic health reviews. In other words, the higher frequency of visits by patients with these diseases would affect the likelihood that associated diseases were diagnosed (i.e., observer bias). To avoid this bias, future studies should be based on the entire population rather than solely on those users of the primary care services.

It is important to note that this work focused on chronic diseases. Although this may ensure comparability with other large studies across different international contexts, future research should consider including the whole range of diseases seen in patients. This would be especially true where the boundaries between “chronic” and “acute” diseases are not always clear, as was demonstrated by Starfield [58]. Often, the recurrence of acute diseases causes them to be chronic, and they should be regarded as chronic diseases in all of their dimensions [58].

Although the different age groups were defined according to the expected biological homogeneity of individuals among groups, the selected thresholds could have influenced the nature of the obtained multimorbidity patterns. The use of different and/or narrower age groups may be advisable in future studies.

To conclude with the potential limitations of this study, it should be noted that this study focused on the occurrence and the simultaneous association of diseases that were defined and registered from the perspective of the professionals caring for the patient and not by the patient him/herself. It is possible that many of the disease manifestations affecting the quality of life of the patient are not met by the defined criteria. Understanding the diseases from this perspective would require complementary methodological approaches that are beyond the scope of this work.

### Comparison with other studies

It is difficult to compare these results with those of other studies because of differences in the disease inclusion criteria, the study populations and the data sources used. This study was also the first to extend the analysis to age groups under 65. The study by Schäfer et al. [29], which was also based on a factor analysis of clinical-administrative data for the German primary care population over 65 years of age, identified three multimorbidity patterns for both men and women: 1) cardiovascular and metabolic disease-related, 2) anxiety, depression, somatoform disorders and pain-related and 3) neuropsychiatric disorder-related. Palomo et al. evaluated the associations between hypothyroidism and various diseases in the Spanish population treated in primary care [9], and these associations were found to precisely conform to the thyroid pattern noted in this study. In the study by Holden et al. based on surveys of Australian workers [59], it was concluded that different health problems are grouped beyond their physiological link with an organ or system. Britt et al. described multimorbidity patterns in the Australian population attended by primary care physicians [14] using the Cumulative Illness Rating Scale (i.e., the CIRS) for the categorisation of clinical entities. Marengoni et al. used a different statistical technique (i.e., cluster analysis) to analyse clusters of diseases in people older than 76 years of age [3]. This technique was also used in the study by Cornell et al. [53], which identified six patterns of multimorbidity among veterans of the United States. These referenced results largely coincide with those from this study and support the existence of the following multimorbidity patterns: mechanical obesity-related, metabolic, neurovascular, liver disease-related, dual diagnosis-driven (psychiatric-substance abuse); and anxiety and depression-related.

### Implications for health systems

Despite the significance of multimorbidity, especially in older individuals, the scientific evidence provided by the clinical practice guidelines is not appropriate for patients with multiple diseases [60], and there is a clear gap in the design of specific intervention programmes for the management of these patients [4]. One of the main consequences of multimorbidity is polypharmacy, which entails the consequent risk for drug interactions and side effects [61]. Accordingly, this study observed examples of these phenomena, including the possible relationship between chronic liver disease and the treatment of ischemic heart disease with statins in men aged 45–64 [26] and the possible interaction between the use of antipsychotics for the behavioural alterations of dementia and the onset of Parkinsonism after the age of 65 [47].

From the standpoint of healthcare management, strong and developed primary care is the best solution to the complex needs of patients with multimorbidity [62]. However, the fragmentation of the current healthcare model hinders integrated and coordinated care between the primary and specialised care levels and between the healthcare and social sectors, which leads to inefficiencies in the care of patients with multimorbidity [62].

Moreover, the importance of other approaches beyond those based on health services, which are needed to ensure the health of the population, should be stressed. For example, the promotion of a healthy lifestyle is closely linked to a decline in the incidence of obesity [63], and as this study has shown, obesity was present in three of the patterns described (cardio-metabolic, mechanical-obesity-thyroidal and psychiatric-substance abuse).

Finally, a change in the focus of etiologic research is required for the study of diseases and their relationship to joint presentations, and an analysis of underlying factors, including pathophysiological, genetic, iatrogenic and/or socioeconomic factors should be promoted.

### Conclusion and future research

The results of this work have demonstrated that the existence of non-random associations between chronic diseases is a reality for the entire population, not only the elderly.

These disease associations give rise to clinically consistent multimorbidity patterns that affect a significant proportion of the population. Most importantly, when studying the lifetime disease process, there are underlying pathophysiological phenomena upon which action can be taken both from a clinical, individual-level perspective and from a public health or population-level perspective.

Knowledge regarding the main disease patterns and their evolution with age should facilitate the design of tools, such as clinical practice guidelines, treatment protocols for patients with multimorbidity and healthcare information systems with alarm signals to monitor for the duplication of visits and diagnostic tests, the incidence of polypharmacy and potential adverse drug reactions, inappropriate hospitalisations and even mortality.

All information available in the health systems should be properly managed to maximise the individual attention given to these patients and to ensure the efficient use of resources. This will positively impact the overall health of the population.

The integration of information is a basic strategy that should be accompanied by structural and functional measures that promote coordination between different actors and levels of the care and that ensure safe and adequate communication among the professionals who care for such patients.

Multimorbidity is a public health problem that must be considered by the planning and organisational frameworks of the health system. This work aimed to provide clinically relevant information to improve this process.

## Supporting Information

Table S1 **Prevalence (%) of chronic EDCs (Expanded Diagnosis Cluster) within each age and sex group.**
(DOCX)
