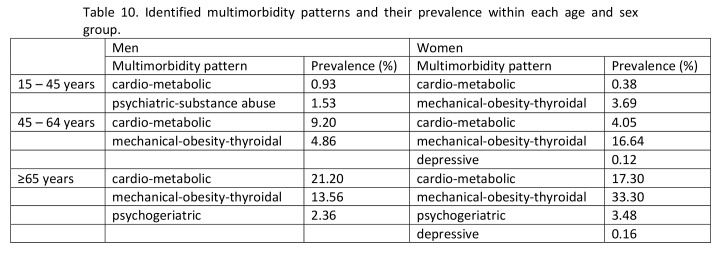# Correction: Multimorbidity Patterns in Primary Care: Interactions among Chronic Diseases Using Factor Analysis

**DOI:** 10.1371/annotation/8c3f813c-8a65-453e-a473-6e3c77093262

**Published:** 2013-12-16

**Authors:** Alexandra Prados-Torres, Beatriz Poblador-Plou, Amaia Calderón-Larrañaga, Luis Andrés Gimeno-Feliu, Francisca González-Rubio, Antonio Poncel-Falcó, Antoni Sicras-Mainar, José Tomás Alcalá-Nalvaiz

In Table 10, some of the prevalence numbers are incorrect. The correct version of Table 10 can be viewed here: 

**Figure:**