# Supplementary material for: Multimorbidity Patterns in Primary Care: Interactions among Chronic Diseases Using Factor Analysis
Source: PLoS One. 2012 Feb 29;7(2):e32190. doi: 10.1371/journal.pone.0032190 (PMC3290548; doi:10.1371/journal.pone.0032190)
Supplement: Table S1 — Prevalence (%) of chronic EDCs (Expanded Diagnosis Cluster) within each age and sex group. (DOCX) [file pone.0032190.s001.docx]

**SUPPORTING INFORMATION**

Table S1. Prevalence (%) of chronic EDCs (Expanded Diagnosis Cluster) within each age and sex group.

|  | Men | | | Women | | |
| --- | --- | --- | --- | --- | --- | --- |
|  | 15 – 44 years | 45 – 64 years | ≥65 years | 15 – 44 years | 45 – 64 years | ≥65 years |
| Low back pain | **9.02** | **13.02** | **13.44** | **10.35** | **17.19** | **18.03** |
| Anxiety, neuroses | **6.69** | **8.85** | **6.63** | **11.69** | **18.53** | **15.18** |
| Disorders of lipoid metabolism | **4.54** | **19.25** | **17.69** | **2.21** | **16.22** | **20.31** |
| Arthropathy | **4.47** | **9.32** | **13.65** | **4.82** | **14.81** | **21.55** |
| Dermatitis and eczema | **4.08** | **5.14** | **7.76** | **4.75** | **5.47** | **6.67** |
| Cervical pain syndromes | **2.39** | **3.12** | **2.79** | **4.43** | **6.15** | **3.82** |
| Asthma | **2.28** | **1.31** | **1.27** | **2.39** | **2.47** | **2.87** |
| Hypertension | **1.83** | **20.80** | **40.95** | **1.06** | **15.72** | **45.86** |
| Obesity | **1.75** | **3.41** | **3.12** | **2.55** | **5.49** | **4.96** |
| Substance use | **1.51** | **1.98** | 0.83 | 0.29 | 0.42 | 0.12 |
| Thyroid disease | **1.24** | **2.33** | **2.81** | **4.33** | **9.26** | **7.22** |
| Diabetes | **1.09** | **10.22** | **19.72** | 0.75 | **5.16** | **14.83** |
| Schizophrenia and affective psychosis | **1.01** | **1.01** | 0.43 | 0.59 | 0.93 | 0.54 |
| Behaviour problems | 0.99 | 0.90 | **1.19** | **1.75** | **1.80** | **2.02** |
| Renal calculi | 0.83 | **1.55** | **1.03** | 0.75 | 0.93 | 0.61 |
| Gastro-oesophageal reflux | 0.83 | **1.80** | **2.40** | 0.58 | **2.10** | **3.37** |
| Disease of hair and hair follicles | 0.80 | 0.21 | 0.20 | **1.28** | 0.96 | 0.86 |
| Haematologic disorders, other | 0.78 | **1.52** | **2.64** | 0.77 | **1.31** | **2.14** |
| Psoriasis | 0.67 | **1.13** | **1.06** | 0.47 | 0.81 | 0.60 |
| Seizure disorder | 0.52 | 0.51 | 0.52 | 0.44 | 0.40 | 0.33 |
| Deafness, hearing loss | 0.47 | **1.13** | **1.77** | 0.34 | 0.89 | **1.77** |
| Chronic liver disease | 0.47 | **1.49** | 0.96 | 0.17 | 0.78 | 0.79 |
| Peripheral neuropathy, neuritis | 0.46 | **1.03** | **1.37** | 0.91 | **2.46** | **1.83** |
| Cardiovascular disorders, other | 0.44 | **1.07** | **2.55** | 0.52 | **1.34** | **2.93** |
| Depression | 0.43 | 0.86 | 0.74 | 0.86 | **2.18** | **1.97** |
| Varicose veins of lower extremities | 0.40 | **1.42** | **2.24** | **1.75** | **4.93** | **7.58** |
| Personality disorders | 0.39 | 0.22 | 0.09 | 0.32 | 0.32 | 0.14 |
| Iron deficiency, other deficiency anaemia | 0.31 | 0.92 | **4.47** | **4.38** | **4.26** | **6.05** |
| Cardiac arrhythmia | 0.29 | **1.42** | **5.88** | 0.17 | 0.75 | **5.04** |
| Other endocrine disorders | 0.28 | 0.69 | 0.75 | 0.94 | **1.73** | **1.49** |
| Irritable bowel syndrome | 0.28 | 0.37 | 0.30 | 0.32 | 0.57 | 0.66 |
| Kyphoscoliosis | 0.28 | 0.08 | 0.08 | 0.41 | 0.30 | 0.31 |
| Generalised atherosclerosis | 0.26 | **1.19** | **3.28** | 0.20 | 0.45 | **1.24** |
| Inflammatory bowel disease | 0.22 | 0.24 | 0.20 | 0.20 | 0.19 | 0.13 |
| Dementia and delirium | 0.21 | 0.62 | **4.04** | 0.15 | 0.82 | **6.41** |
| Glaucoma | 0.18 | **1.37** | **3.49** | 0.16 | **1.62** | **4.07** |
| Tuberculosis infection | 0.18 | 0.12 | 0.16 | 0.15 | 0.11 | 0.13 |
| Disorders of the immune system | 0.18 | 0.26 | 0.25 | 0.27 | 0.45 | 0.34 |
| Prostatitis | 0.18 | 0.53 | 0.66 | 0.02 | 0.02 | 0.00 |
| Gout | 0.17 | **1.39** | **2.02** | 0.00 | 0.08 | 0.32 |
| Attention deficit disorder | 0.17 | 0.02 | 0.03 | 0.05 | 0.01 | 0.01 |
| Low impact malignant neoplasms | 0.17 | 0.93 | **2.42** | 0.35 | **1.88** | **2.52** |
| Neurologic disorders, other | 0.16 | 0.38 | 0.48 | 0.20 | 0.61 | 0.55 |
| Blindness | 0.13 | 0.29 | 0.32 | 0.14 | 0.24 | 0.36 |
| Emphysema, chronic bronchitis, COPD | 0.12 | **2.34** | **9.57** | 0.11 | 0.78 | **2.42** |
| Prostatic hypertrophy | 0.12 | **4.83** | **15.08** | -- | 0.01 | 0.03 |
| Thrombophlebitis | 0.10 | 0.34 | 0.78 | 0.12 | 0.38 | 1.00 |
| Developmental disorder | 0.10 | 0.08 | 0.01 | 0.11 | 0.05 | 0.02 |
| Cerebrovascular disease | 0.09 | 0.96 | **3.78** | 0.06 | 0.46 | **2.67** |
| Retinal disorders (excluding diabetic retinopathy) | 0.09 | 0.48 | 0.82 | 0.04 | 0.37 | 0.86 |
| Acute myocardial infarction | 0.08 | **1.75** | **3.21** | 0.01 | 0.19 | 0.93 |
| Chronic ulcer of the skin | 0.07 | 0.27 | **1.14** | 0.03 | 0.15 | **1.67** |
| Ischemic heart disease (excluding acute myocardial infarction) | 0.07 | **1.86** | **5.52** | 0.02 | 0.40 | **2.78** |
| Cardiac valve disorders | 0.06 | 0.36 | 0.76 | 0.05 | 0.22 | 0.95 |
| Multiple sclerosis | 0.06 | 0.09 | 0.05 | 0.09 | 0.15 | 0.04 |
| Osteoporosis | 0.05 | 0.45 | **1.18** | 0.13 | **8.89** | **13.26** |
| HIV, AIDS | 0.05 | 0.06 | 0.01 | 0.06 | 0.02 | 0.00 |
| High impact malignant neoplasms | 0.05 | 0.06 | 0.04 | 0.05 | 0.04 | 0.04 |
| Cataract, aphakia | 0.05 | 0.89 | **6.03** | 0.05 | 0.86 | **7.28** |
| Malignant neoplasms, colorectal | 0.05 | 0.51 | **1.42** | 0.05 | 0.35 | 0.77 |
| Nephritis, nephrosis | 0.04 | 0.12 | 0.16 | 0.05 | 0.06 | 0.13 |
| Respiratory disorders, other | 0.04 | 0.08 | 0.20 | 0.05 | 0.10 | 0.12 |
| Chromosomal anomalies | 0.03 | 0.02 | 0.01 | 0.04 | 0.01 | 0.00 |
| Diverticular disease of colon | 0.03 | 0.29 | 0.77 | 0.02 | 0.33 | **1.23** |
| Haemolytic anaemia | 0.03 | 0.02 | 0.04 | 0.05 | 0.05 | 0.05 |
| Paralytic syndromes, other | 0.03 | 0.03 | 0.05 | 0.02 | 0.03 | 0.03 |
| Congenital heart disease | 0.03 | 0.03 | 0.01 | 0.04 | 0.03 | 0.03 |
| Congestive heart failure | 0.03 | 0.28 | **1.83** | 0.02 | 0.12 | **2.34** |
| Spinal cord injury/disorders | 0.02 | 0.02 | 0.11 | 0.02 | 0.01 | 0.06 |
| Pulmonary embolism | 0.01 | 0.07 | 0.19 | 0.02 | 0.03 | 0.22 |
| Parkinson's disease | 0.01 | 0.19 | **1.25** | 0.01 | 0.23 | 0.99 |
| Malignant neoplasms, lymphomas | 0.01 | 0.03 | 0.05 | 0.01 | 0.03 | 0.05 |
| Malignant neoplasms, bladder | 0.01 | 0.29 | 0.81 | -- | 0.05 | 0.11 |
| Malignant neoplasms of the skin | 0.01 | 0.05 | 0.21 | 0.01 | 0.05 | 0.08 |
| Malignant neoplasms, stomach | 0.01 | 0.06 | 0.14 | 0.00 | 0.02 | 0.07 |
| Utero-vaginal prolapse | 0.00 | -- | 0.01 | 0.08 | 0.56 | 0.88 |
| Hypospadias, other penile anomalies | 0.00 | 0.00 | 0.00 | -- | -- | 0.00 |
| Chronic cystic disease of the breast | 0.00 | 0.01 | 0.01 | 0.47 | 0.73 | 0.26 |
| Malignant neoplasms, prostate | 0.00 | 0.43 | **3.06** | -- | -- | 0.00 |
| Malignant neoplasms, breast | -- | 0.00 | -- | 0.04 | 0.28 | 0.21 |
| Malignant neoplasms, cervix, uterus | -- | -- | -- | 0.02 | 0.04 | 0.01 |
| Malignant neoplasms, kidney | -- | 0.02 | 0.05 | 0.00 | 0.01 | 0.01 |
| Malignant neoplasms, lung | -- | 0.06 | 0.07 | 0.00 | 0.02 | 0.02 |
| Malignant neoplasms, pancreas | -- | 0.02 | 0.06 | -- | 0.02 | 0.04 |
| Surgical aftercare | -- | -- | -- | -- | -- | -- |
| Transplant status | -- | -- | -- | -- | -- | -- |
| Cardiomyopathy | -- | -- | -- | -- | -- | -- |
| Generalised atherosclerosis | -- | -- | -- | -- | -- | -- |
| Diabetic retinopathy | -- | -- | -- | -- | -- | -- |
| Endometriosis | -- | -- | -- | -- | -- | -- |
| Chronic pancreatitis | -- | -- | -- | -- | -- | -- |
| Lactose intolerance | -- | -- | -- | -- | -- | -- |
| Peripheral vascular disease | -- | -- | -- | -- | -- | -- |
| Aortic aneurysm | -- | -- | -- | -- | -- | -- |
| Inherited metabolic disorders | -- | -- | -- | -- | -- | -- |
| Vesicoureteral reflux | -- | -- | -- | -- | -- | -- |
| Haemolytic anaemia | -- | -- | -- | -- | -- | -- |
| Aplastic anaemia | -- | -- | -- | -- | -- | -- |
| Malignant neoplasms, ovary | -- | -- | -- | -- | -- | -- |
| Malignant neoplasms, oesophagus | -- | -- | -- | -- | -- | -- |
| Malignant neoplasms, liver and biliary tract | -- | -- | -- | -- | -- | -- |
| Acute leukaemia | -- | -- | -- | -- | -- | -- |
| Congenital hip dislocation | -- | -- | -- | -- | -- | -- |
| Congenital anomalies of limbs, hands and feet | -- | -- | -- | -- | -- | -- |
| Cervical pain syndromes | -- | -- | -- | -- | -- | -- |
| Muscular dystrophy | -- | -- | -- | -- | -- | -- |
| Quadriplegia and paraplegia | -- | -- | -- | -- | -- | -- |
| Cerebral palsy | -- | -- | -- | -- | -- | -- |
| Cleft lip and palate | -- | -- | -- | -- | -- | -- |
| Chronic renal failure | -- | -- | -- | -- | -- | -- |
| Renal disorders, other | -- | -- | -- | -- | -- | -- |
| Cystic fibrosis | -- | -- | -- | -- | -- | -- |
| Sleep apnoea | -- | -- | -- | -- | -- | -- |
| Tracheostomy | -- | -- | -- | -- | -- | -- |

Note: a prevalence ≥1% has been highlighted in bold.
